# New insights into HCV replication in original cells from *Aedes* mosquitoes

**DOI:** 10.1186/s12985-017-0828-z

**Published:** 2017-08-22

**Authors:** Catherine Fallecker, Alban Caporossi, Yassine Rechoum, Frederic Garzoni, Sylvie Larrat, Olivier François, Pascal Fender, Patrice Morand, Imre Berger, Marie-Anne Petit, Emmanuel Drouet

**Affiliations:** 1 Institut de Biologie Structurale (IBS), Université Grenoble Alpes, CEA, CNRS, 71 Avenue des Martyrs, 38000 Grenoble, France; 2 Institut de Biologie et de Pathologie (IBP), Centre Hospitalier Universitaire (CHU) Grenoble-Alpes, CS10217, 38043 Grenoble Cedex 9, France; 3 Laboratoire Techniques de l’Ingénierie Médicale et de la Complexité, UMR CNRS 5525, Université Grenoble-Alpes, Grenoble, France; 4 EMBL Grenoble, 71 Avenue des Martyrs, CS90181, 38042 Grenoble Cedex 9, France; 5 The School of Biochemistry, University of Bristol, University Walk, Bristol, Clifton BS8 1TD UK; 6 Centre de Recherche en Cancérologie de Lyon (CRCL), UMR INSERM 1052/CNRS 5286, 151 Cours Albert Thomas, 69424 Lyon, Cedex 03 France; 7 Present Address: Dan L Duncan Cancer Center, Baylor College of Medicine, One Baylor Plaza, Houston, TX USA

**Keywords:** Hepatitis C virus (HCV), HCV pseudo particles, *Aedes aegypti*, *Aedes albopictus*, Human hepatocytes

## Abstract

**Background:**

The existing literature about HCV association with, and replication in mosquitoes is extremely poor. To fill this gap, we performed cellular investigations aimed at exploring (i) the capacity of HCV E1E2 glycoproteins to bind on *Aedes* mosquito cells and (ii) the ability of HCV serum particles (HCVsp) to replicate in these cell lines.

**Methods:**

First, we used purified E1E2 expressing baculovirus-derived HCV pseudo particles (bacHCVpp) so we could investigate their association with mosquito cell lines from *Aedes aegypti* (Aag-2) and *Aedes albopictus* (C6/36). We initiated a series of infections of both mosquito cells (*Ae aegypti* and *Ae albopictus*) with the HCVsp (Lat strain - genotype 3) and we observed the evolution dynamics of viral populations within cells over the course of infection *via* next-generation sequencing (NGS) experiments.

**Results:**

Our binding assays revealed bacHCVpp an association with the mosquito cells, at comparable levels obtained with human hepatocytes (HepaRG cells) used as a control. In our infection experiments, the HCV RNA (+) were detectable by RT-PCR in the cells between 21 and 28 days post-infection (p.i.). In human hepatocytes HepaRG and *Ae aegypti* insect cells, NGS experiments revealed an increase of global viral diversity with a selection for a quasi-species, suggesting a structuration of the population with elimination of deleterious mutations. The evolutionary pattern in *Ae albopictus* insect cells is different (stability of viral diversity and polymorphism).

**Conclusions:**

These results demonstrate for the first time that natural HCV could really replicate within *Aedes* mosquitoes, a discovery which may have major consequences for public health as well as in vaccine development.

**Electronic supplementary material:**

The online version of this article (doi:10.1186/s12985-017-0828-z) contains supplementary material, which is available to authorized users.

## Background

Hepatitis C Virus (HCV) is a member of the Hepacivirus genus which belongs to the *Flaviviridae* family. It is an enveloped single stranded RNA virus which is present worldwide [1]. Most of the Flaviviruses are causative agents for major epidemic or endemic diseases including Yellow Fever (YF), Dengue Fever (DEN), West Nile Fever (WN), and recently Zika Virus Disease [2, 3]. Most of these viruses are transmitted by vectors in very different epidemiological ways. Some diseases are typically human (or linked to primates) and do not affect animals (e.g. DEN). Others are zoonotic and affect humans accidentally, e.g. Japanese Encephalitis, Saint-Louis Encephalitis and WN. Finally, certain Flaviviruses can circulate in epidemic form both in human and animal populations (e.g. YF). These different epidemiological modes of transmission share in common viral amplification in insect cells, therefore the denomination ‘arbovirus’ [4].

HCV is a severe pathogen, giving rise to liver inflammation and causing acute or chronic disease. New drugs targeting HCV are now becoming available, but notwithstanding, HCV infected 180 million people worldwide in 2013 [5]. Attempts to develop a prophylactic vaccine against HCV which could prevent infection have largely failed to date [5]. HCV was identified 30 years ago, but its origin remains elusive. HCV is a blood-borne virus and the epidemic appears to have been fueled by new parenteral transmission routes associated with blood transfusions, immunization, and more recently intravenous drug abuse [6]. The immediate source of HCV associated with its pandemic spread has been identified to the areas of the central and west sub-Saharan Africa [7], as well as south and south-east Asia, where genetically diverse variants of HCV appear to have circulated for several hundred years [8].

**Fig. 1:**
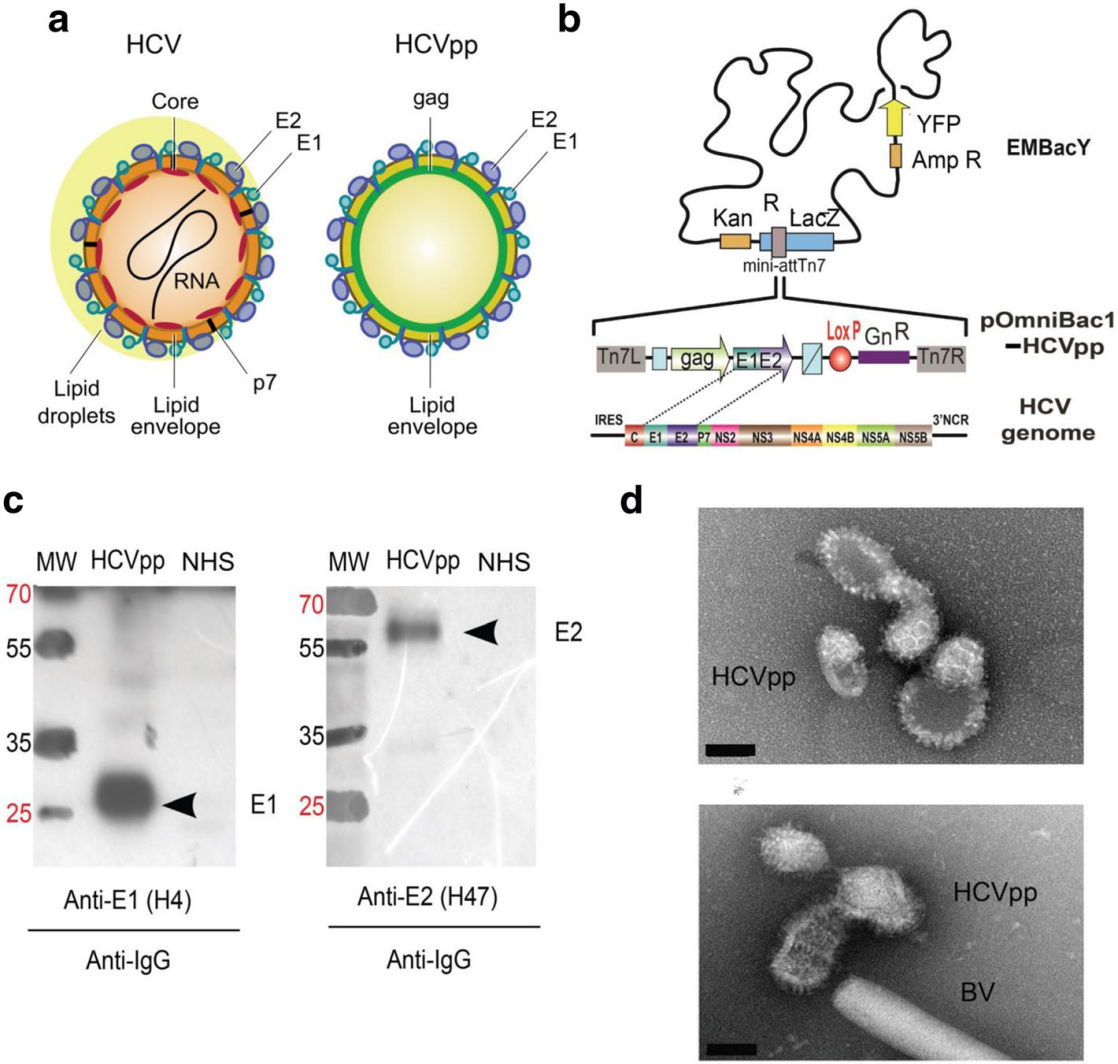
MultiBac-based HCV pseudo particles (*bac*HCVpp). **a** Hepatitis C virus (HCV) and HCV pseudo particles (HCVpp) are shown in a schematic representation. Glycoprotein E1 is colored in green, glycoprotein E2 in blue, HCV core in red, HIV gag in light green. HCV in contrast to HCVpp contains also ion channel proteins (p7), lipid droplets on the surface and an RNA genome. **b** HCVpp was produced with the MultiBac system. A transfer plasmid (pOmniBac1-HCVpp) was used, containing expression cassettes encoding for HCV E1E2 precursor fusion protein on one hand, and for HIV gag protein to initiate budding or particles on the other. The EmBacY baculoviral genome was used to infect Sf21 insect cell cultures for HCVpp production. E1E2, glycoprotein precursor fusion; gag, HIV gag protein; YFP, yellow fluorescent protein; Amp, ampicillin resistance marker; Kan, kanamycin resistance marker, Gn, gentamycin resistance marker; LacZ, blue/white selection marker to identify recombinant baculoviral genome; mini-attTn7, attachment site for integration of heterologous genes by Tn7 transposition; Tn7L and Tn7R, DNA recognition sequences for Tn7 transposase; LoxP, inverted imperfect repeat for Cre-LoxP DNA fusion; IRES, internal ribosomal entry site; 3′NCR, 3′ non-coding region. C, E1, E2, P7, NS2, 3, 4A, 4B, 5A, 5B are HCV proteins. **c** Results from Western Blot experiments are shown, evidencing HCV E1 and E2 proteins in purified *bac*HCVpp. MW, Molecular Weight marker; Anti-E1 (H4), primary antibody specific for E1; Anti-E2 (H47), antibody specific for E2; NHS (Normal Human Serum). Alkaline phosphatase coupled Anti-IgG was used as secondary antibody. Molecular weights (in kDa) of marker bands are indicated. **d** Negative-stain electron micrographs of MultiBac-produced HCVpp (*bac*HCVpp) are shown. BV denotes baculovirions. Scale bar, 50 nm

Many different in vitro models have been developed to investigate HCV. For example, virus-like particles (VLPs) comprising HCV core proteins and the E1E2 heterodimeric envelope glycoproteins were produced in insect cells [9] and used for immunization of chimpanzees [10]. Furthermore, rhabdoviral (Vesicular Stomatitis Virus, VSV) [11] and retroviral (Lentivirus or Murine Leukemia Virus) systems have been utilized to obtain pseudotypes or so-called HCV pseudo particles (HCVpp) from mammalian cells [12]. These mammalian-cell derived pseudo particles have been instrumental for characterizing HCV specific neutralizing antibodies [13]. In contrast to HCV-VLPs, HCVpps are made of a heterologous core formed by a retroviral protein (e.g. gag), and display the HCV E1E2 proteins on their surface (Fig. 1a) [9]. HCVpp, similar to HCV-VLPs, cannot undergo a complete viral life cycle, notably as they are replication incompetent for lack of viral genomic RNA [14]. These particles are highly useful for studying HCV binding and entry, as shown in Huh-7 cells by means of GFP-labeling approaches [15]. Other robust HCV models called HCVcc (HCV “cell cultured”) have been developed. This system based initially on transfection of HCV-JFH1 clone genotype 2a RNA in the human hepatoma cell line (Huh-7 and derivatives) has demonstrated its efficiency to produce complete infectious viral particles by infection route [14].

Several HCV risk factors have been defined in the context of the spreading epidemic and its possible control. Thus, the reservoir was described as only human and more generally primates, and the HCV contamination route was assumed to be mainly through blood contact. However, for a non-negligible proportion (more than 20%) of HCV infections the route of transmission could not be established with confidence [16]. In some cases, especially regarding the endemic HCV transmission, alternative routes for spreading HCV could exist. This hidden HCV epidemic, notably in the USA, represents a significant socio-economic damage potential, causing considerable alarm [17]. As a consequence, investigations on HCV transmission modes merit further consideration. Taken together, the currently available results regarding the spread of HCV remain contradictory and their interpretation is a matter of intense debate [18–21]. In 2001, we demonstrated previously that the AP61 mosquito cell line from *Aedes pseudoscutellaris* could associate with HCV particles [22]. We set out here to investigate alternative transmission routes for HCV that could account for the hidden HCV epidemic. For this purpose, we performed cellular investigations; first we explored the binding step of HCV by using baculovirus-derived HCV pseudo particles (*bac*HCVpp) [23, 24]; second, we engaged a series of infections of both mosquito cells (*Ae aegypti* and *Ae albopictus*) with serum-derived HCV particles (Lat strain of genotype 3) [25] and we followed up the evolution dynamics of viral populations within cells over the course of infection, through next-generation sequencing (NGS) experiments.

## Methods

### Cell cultures

HepaRG cells were cultured as previously described [25, 26] and using HepaRG® Culture kit for HCV infection (catalog number: KIT902, Biopredic International). *Aedes albopictus* C6/36 cells were grown in Leibovitz L15 medium and *Aedes aegypti* Aag-2 cells in Schneider medium (GIBCO Life Technologies). Differentiated HepaRG cells were seeded in a 6-well tissue culture (TC) plate and grown in monolayer for two weeks [25]. Mosquito cells were plated after four passages in 4-well Lab-Tek TC plates. The *Aedes aegypti* Ktmos1 cells were generated as previously described [27] and cultivated in Leibovitz L15 growth medium supplemented with 20% fetal bovine serum, 1% penicillin- streptomycin, 1% L-glutamine (see Additional file 1 and Additional files 2 and 3: Figure S1 and S2).

### MultiBac-based production of HCV pseudo particles (*bac*HCVpp)

Recombinant *bac*HCVpp were produced using the MultiBac system we developed for multiprotein applications [23]. HCV E1E2 envelope proteins and HIV gag protein were cloned into transfer plasmid pOmniBac1 [28] and inserted via Tn7 transposition into the EmBacY baculoviral genome [23, 28]. Transfection, virus amplification and recombinant *bac*HCVpp expression were performed following established standard operating procedures [24]. Briefly, *bac*HCVpp were produced in 250 mL of Sf21 (*Spodoptera frugiperda)* cells cultured in suspension in serum-free medium. A gene encoding for yellow fluorescent protein (YFP) is present in the EMBacY baculoviral genome, to be used as a reporter for virus performance and recombinant protein production. When YFP fluorescence reached a plateau, the supernatant was collected, cleared from cells and cell debris by centrifugation and filtration, and pelleted by ultracentrifugation at 130,000 g overnight in a swinging bucket rotor. The *bac*HCVpp pellet was resuspended in phosphate-buffered saline (PBS) and loaded on a 20–60% continuous sucrose gradient. *Bac*HCVpp was apparent in the gradient as a unique, visible band and was collected. Purified *bac*HCVpp was analyzed by negative-stain electron microscopy (EM) to verify structural integrity. Recombinant baculovirus-derived Influenza Virus pseudo particles (*bac*Flupp) were also produced as controls (with M1 matrix protein instead of HIV gag core protein) (Additional file 4: Figure S3)

### Cell binding assay

Binding assays were performed when the cultures each reached confluence, with *bac*HCVpp and also with *bac*Flupp, both produced by using the MultiBac system [23]. The binding experiments were performed with 20 μl of particles (OD_650nm_ = 0.2). Incubation was for two hours at 37 °C for the human HepaRG cell culture, and at 28 °C for the mosquito cell line (Ktmos 1 and C6/36) cultures. Cells were washed twice with their respective media before further analysis.

### Immunofluorescence (IF) assay

Cells were fixed with 2% paraformaldehyde (PFA) for 20 min at 37 °C for the human HepaRG cells and 28 °C for the both mosquito cell lines, respectively, in the TC plates. Each well was washed three times with PBS 1×. Immunofluorescence (IF) assays were performed by incubation for one hour at 37 °C or 28 °C, respectively, of each well of the TC plates with anti-E1 (clone H4) mouse IgG (hybridoma supernatant diluted 1/5) as the primary antibody. The anti-E1 mouse IgGs which reacted with *bac*HCVpp bound to cells were then detected by incubation for one hour with Cy3-coupled goat anti-mouse IgG (Sigma–Aldrich), at a dilution of 1/1000 using a motorized inverted Olympus IE81 microscope and differential interference contrast (DIC).

### Infection experimental protocols

An amount of 50,000 cells has been seeded in Lab-Tek and 24-well plates with 100,000 cells/mL under a volume of 500 μl. Insect cells and HepaRG cells were incubated at 28 °C and 37 °C respectively. HCV infection experiments were carried out using purified HCV serum particles (HCVsp Lat strain, genotype 3a), which contained 6 × 10^7^ copies of HCV RNA per mL (Amplicor HCV Monitor test, Roche diagnostics, Meylan, France). HCVs Lat strain was previously well-characterized and used for binding/infection experiments (see Additional file 1: Table S1) [26] [25]. HepaRG and C6/36 cells were inoculated 3 days postplating (p.p.), whereas Ktmos1 cells were infected 20 days p.p. All the cells were infected for 18 h at 37 °C (C6/36 and HepaRG cells) and 28 °C (Ktmos1 cells) with 10^5^ copies of HCV RNA corresponding to multiplicity of infection (m.o.i.) of 1. Before infection with the HCVsp, the particles were resuspended in 500 μl of L15 medium (insect cells) or of working HepaRG medium 710 (HepaRG® Culture kit for HCV infection, catalog number: KIT902, Biopredic International, Rennes, France). On day 1 postinfection (p.i.), extensive washings (5 times) of the cells were done. The medium was changed at day 7 p.p. and then each week at days 14, 21 and 28. Human Embryonic Kidney (HEK) 293 cells, which have been shown not to bind HCVpp, were also infected by HCVsp Lat strain. All the experiments of HCV infection were performed in a P3 facility, and according to protocols described in Additional file 5: Figure S4.

### Detection of HCV RNA (+) by one-step reverse transcription polymerase chain reaction

After HCV infection, the cells were pelleted at 800 rpm for 10 min, and all the pellets collected were frozen at −80°. RNA extraction from the cells was performed in the P3 lab, by using the kit RNase easy (Qiagen) and turbo DNase 1 U in a 500 μl volume. Afterwards, the reverse transcription was performed using primers located in the 5′NCR region of all HCV genotypes (5NC1: GCA GAA AGC GTC TAG CCA TGG CGT, and 5NC2: CTC GCA AGC ACC CTA TCA GGC AGT). After a denaturation step, the RNA template was incubated at 42 °C for 50 min with 7,5 U Primescript Reverse, then the cDNA was amplified for 45 cycles. The amplicons were analyzed onto an agarose gel and checked by Sanger sequencing (Additional file 6: Figure S5).

### Investigation of the viral diversity and polymorphism by NGS

The amplicons corresponding to the amplification of cDNA were submitted to a 454 GS FLX. An additional PCR step added adapter and sample-specific multiplex identifiers in forward strand. Forward amplicon sequencing program was then applied. After filtering out reads strictly shorter than 200 bp, identifying reads present more than once in the dataset (multiplicity ≥2) and trimming the remaining 454 primers from them, we extracted from each sample a sub-dataset of reads based on multiplicity in descending order such that we kept at most one hundred sequences per sample, i.e. the most frequent reads observed in each sample. We finally merged sub-datasets obtained and processed them with a multiple sequence alignment algorithm [29] to compare the evolution of “H”, “K” and “C” (respectively for HepaRG, Ktmos1 and C6/36 cells) versus the “Lat” one, the HCV inoculum. We used the R package APE to compute nucleotide diversity and Tajima’s D statistics for multiple alignments in the fast format [30, 31]. Nucleotide diversity is a population genetic estimate of the effective mutation rate, defined as twice the product of the mutation rate by the number of effectively replicating particles or effective population size. An increase in nucleotide diversity indicates that the size of the population increases. Negative values of the Tajima’s D statistic are indicative of positive selection or population expansion after a bottleneck, whereas values close to zero are observed for populations evolving under mutation-drift equilibrium. Positive values of the Tajima’s D statistics are indicative of balancing selection or population divergence. Empirical distributions of nucleotide diversity and Tajima’s D statistic were obtained after considering 200 random multiple alignments from the set all viral sequences. The size of the multiple alignments was equal to the number of haplotypes detected. Phylogenetic analyses were performed using neighbor-joining trees and the F84 model of molecular evolution [32].

## Results

### HCV envelope proteins associate with *Aedes* Mosquito cells lines

**Fig. 2:**
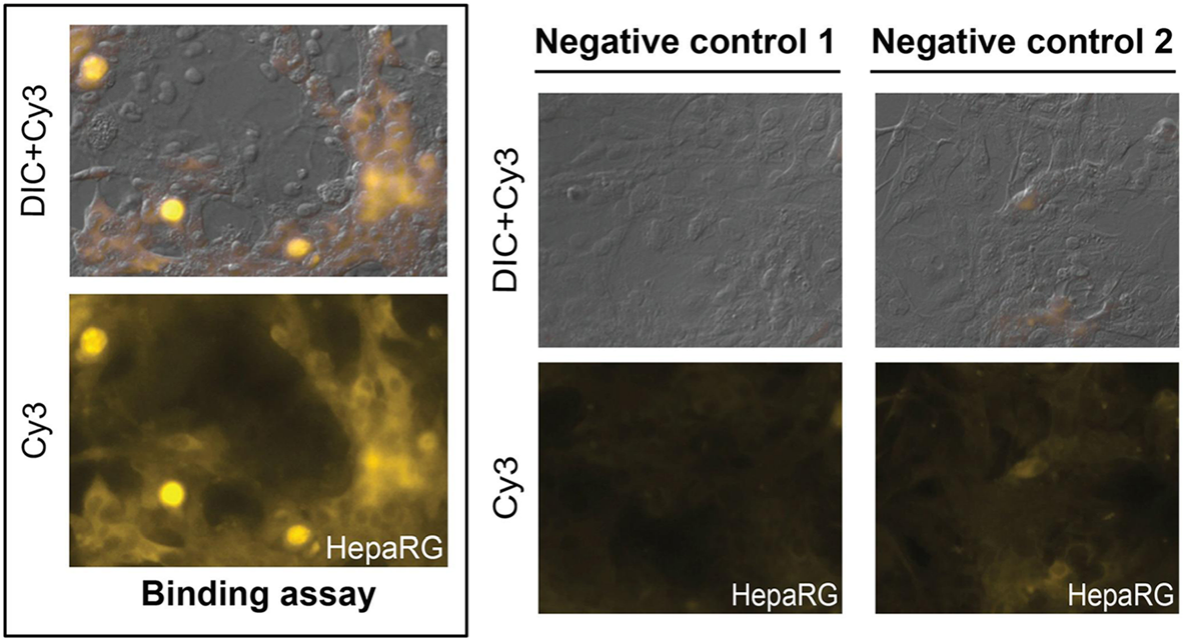
*Bac*HCVpp associate with human hepatocyte cells. Human HepaRG hepatocytes incubated with *bac*HCVpp for two hours are shown (left). Binding assay was performed using primary anti-E1 (H4) monoclonal antibody and visualized with *Cy3*-labeled secondary anti-IgG antibody. Negative controls are also shown (right). Negative control 1, primary antibody was omitted; negative control 2, *bac*Flupp was used instead of *bac*HCVpp. DIC, Differential Interference Contrast

We successfully produced recombinant *bac*HCVpp in Sf21 insect cell cultures infected with a composite baculovirus containing two heterologous expression cassettes, one encoding for the HCV E1E2 precursor fusion protein, and the other encoding for HIV gag protein (Fig. 1b). Then we confirmed authentic processing of the precursor protein and the presence of E1 and E2 on our *bac*HCVpps by Western Blot (WB) of the sedimented particles and specific antibody staining using anti-E1 (H4) and anti-E2 (H47) primary monoclonal antibodies (Fig. 1c). Two strong signals at 21 kDa and 70 kDa were observed, respectively. Negative stain electron microscopic (EM) examination of purified bacHCVpp samples revealed spherical particles decorated with spikes (Fig. 1d). To determine the functionality of our purified *bac*HCVpps, we performed a binding assay with human hepatocytes, HepaRG cells (Fig. 2). These cells were already used for the binding [26] and the infection [25] with serum-derived HCV particles (HCVsp), and thus can be considered as a robust reference model. The results showed that *bac*HCVpps efficiently associate with human HepaRG hepatocytes in a specific manner, consistent with what was observed for HCVsp [26].

**Fig. 3:**
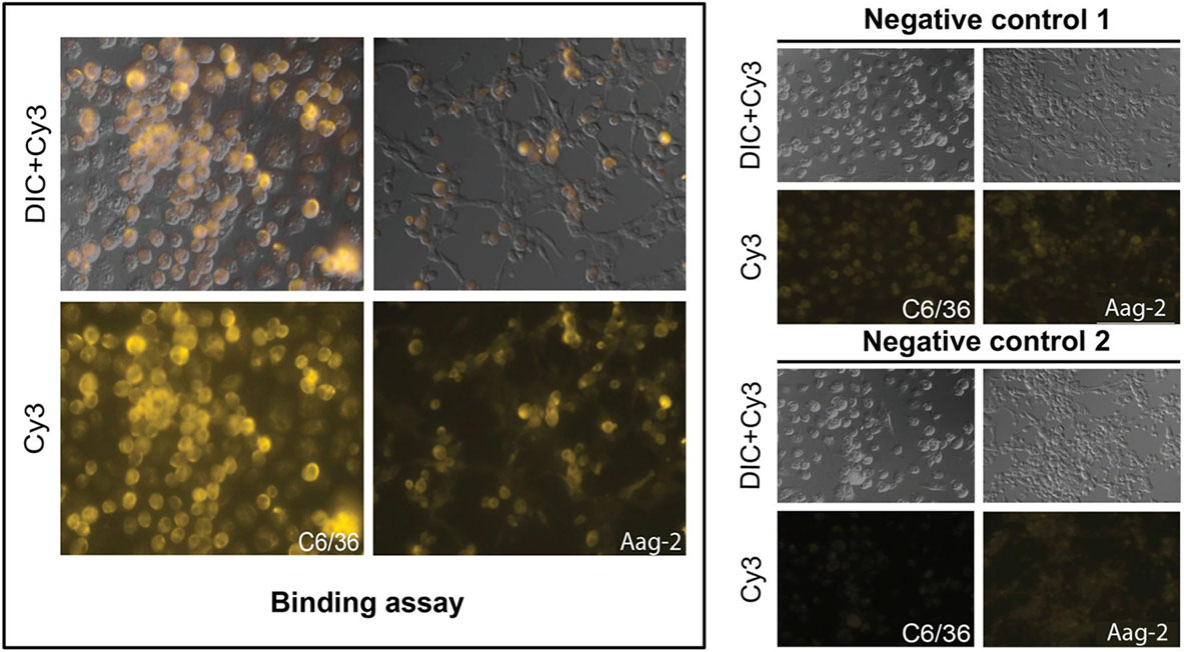
*Bac*HCVpp associate with *Aedes* mosquito cell lines C6/36 and Aag-2. Mosquito cells incubated with *bac*HCVpp for two hours are shown (left). Binding assay was performed using primary anti-E1 (H4) monoclonal antibody and visualized with *Cy3*-labeled secondary anti-IgG antibody. Negative controls are also shown (right). Negative control 1, primary antibody was omitted; negative control 2, *bac*Flupp was used instead of *bac*HCVpp. DIC, Differential Interference Contrast

Having established the E1E2 immunological reactivity, morphological integrity and functionality of our recombinant *bac*HCVpp system, we then asked whether our *bac*HCVpps would likewise associate with mosquito cell lines. We chose *Aedes aegypti* Aag-2 (an *Ae. aegypti* cell lineage of embryonic origin) [33] and *Aedes albopictus* C6/36 cell lines for these experiments. These cell lines were shown to associate with and be permissive to some other Flaviviruses, including Dengue Virus (DEN) and Yellow Fever virus (YF) [33, 34]. As shown in the Fig. 3, we detected clear positive signals for the two mosquito cell lines. The signals observed for the human HepaRG hepatocytes (Fig. 2) and for *Aedes aegypti* Aag-2 were at a comparable level, while a particularly strong signal was detected with *Aedes albopictus* C6/36 cells (Fig. 3).

The specificity of our binding assays were established either by omitting primary antibody (negative control 1, Figs. 2 and 3) or by using a non-HCV *bac*pp derived from Influenza Virus (*bac*Flupp) instead of *bac*HCVpps (negative control 2, Figs. 2 and 3). Taken together, our results showed that the signals obtained with human HepaRG hepatocytes as well as those obtained with the two different mosquito cell lines, *Aedes aegypti* Aag-2 and *Aedes albopictus* C6/36, are specific for *bac*HCVpps. This indicates that the *Aedes* mosquito cell lines are able to recognize HCV envelope proteins (E1, E2 and/or E1E2) expressed on the surface of our *bac*HCVpps, as well as human HepaRG hepatocytes. Then, the question is to know whether these mosquito cells are able to be in vitro infected with HCVsp, as already demonstrated for human HepaRG hepatocytes.

### *Aedes* Mosquito cell lines are susceptible to HCVsp infection

**Fig. 4:**
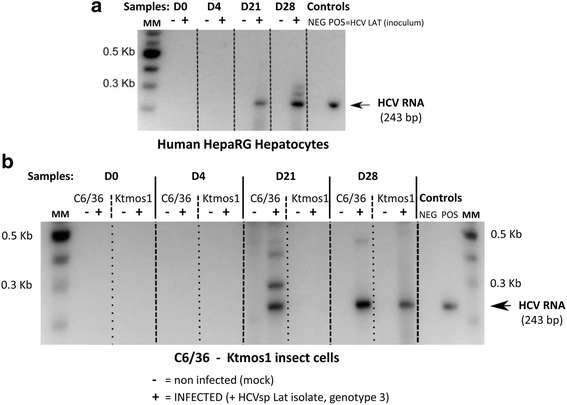
Detection of HCV RNA (+) by one-step Reverse Transcription Polymerase Chain Reaction (RT-PCR). HCV RNA (+) was detected by RT-PCR in non-infected (mock) cells (−) and HCV-infected (+) human HepaRG hepatocytes (**a**) or insect cells (**b**, C6/36 *Ae. Albopictus* and Ktmos1 *Ae. Aegypti* cells) collected at days 0 (D0), 4 (D4), 21 (D21) and 28 (D28) p.i. (samples). The inoculum HCVsp (LAT isolate, genotype 3) was used as positive control (POS) and load in the last lane (A) and just before the last lane (B). MM, molecular weights markers (in Kb). Agarose gels (1%) were represented and showed products resulting from amplification using 5NC1/2NC2 primers. The specific HCV RNA band corresponded to 243 bp

Because we previously showed that HCV envelope was able to bind to *Aedes aegypti* and *Aedes albopictus* cells, we performed infection with HCVsp Lat strain (genotype 3) on *Aedes albopictus* C6/36 cell line and *Aedes aegypti* Ktmos1 cells. The HepaRG cells were used as a reference positive control. As illustrated in Fig. 4, HCV RNA(+) was detected by RT-PCR at days 21 and 28 p.i. for HepaRG cells (Fig. 4a) as well as for the C6/36 mosquito cell line (Fig. 4b), and at day 28 p.i. for *Aedes aegypti* Ktmos1 cells (Fig. 4b). All the cells are thus positive for intracellular HCV RNA at day 28 p.i. We will notice negative results at days 0 and 4 p.i., reflecting production of newly synthesized HCV RNA molecules. The PCR products were then subjected to Sanger sequencing. Mutations were detected within the loops IIb and IIa for HCV-infected HepaRG and Ktmos1 cells and one mutation was observed for HCV-infected C6/36 cells within loop IId of the IRES region of HCV RNA genome (Additional file 5: Figure S4). Eventually, we did not obtain any RT-PCR signals at any day explored (over a period from four days to 28 days) in the HEK 293 experiments (Additional file 7: Figure S6).

### Deciphering a divergent evolution of viral strains in *Aedes aegypti* cells as in human hepatocytes

**Fig. 5:**
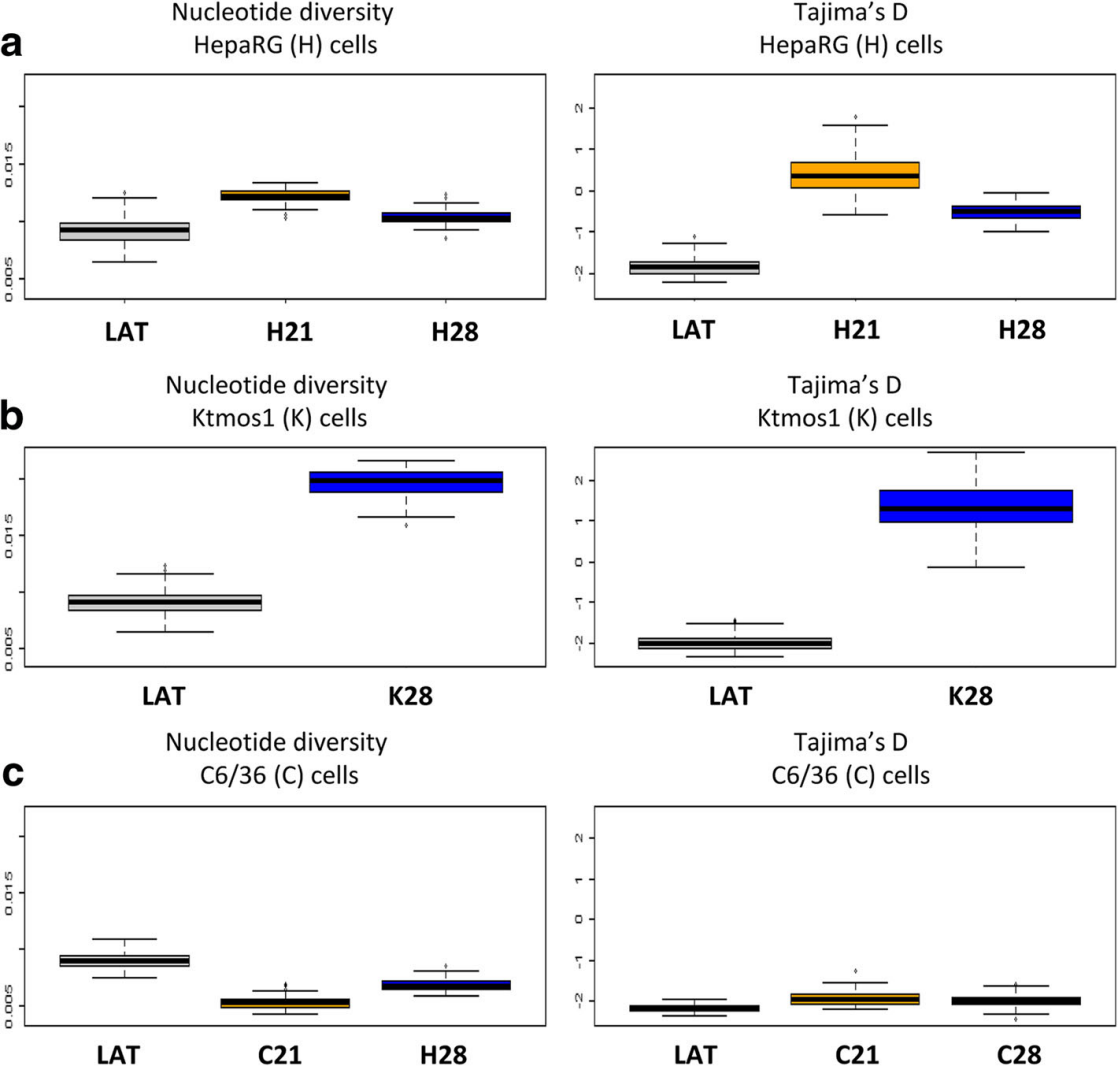
Distributions of nucleotide diversity and Tajima’s D statistics for HCV strains present in the inoculum and HCV-infected cells at days 21 and/or 28 p.i. Nucleotide diversity increases for the strains in HepaRG (**a**, H21/H28) and Ktmos1 (**b**, K28) cells whereas it decreases for the strains in C6–36 (**c**, C21/C28) cells. Tajima’s D increases toward 0 for the strains in HepaRG and Ktmos1 cells whereas it does not evolve for the strains in C6–36 cells

**Fig. 6:**
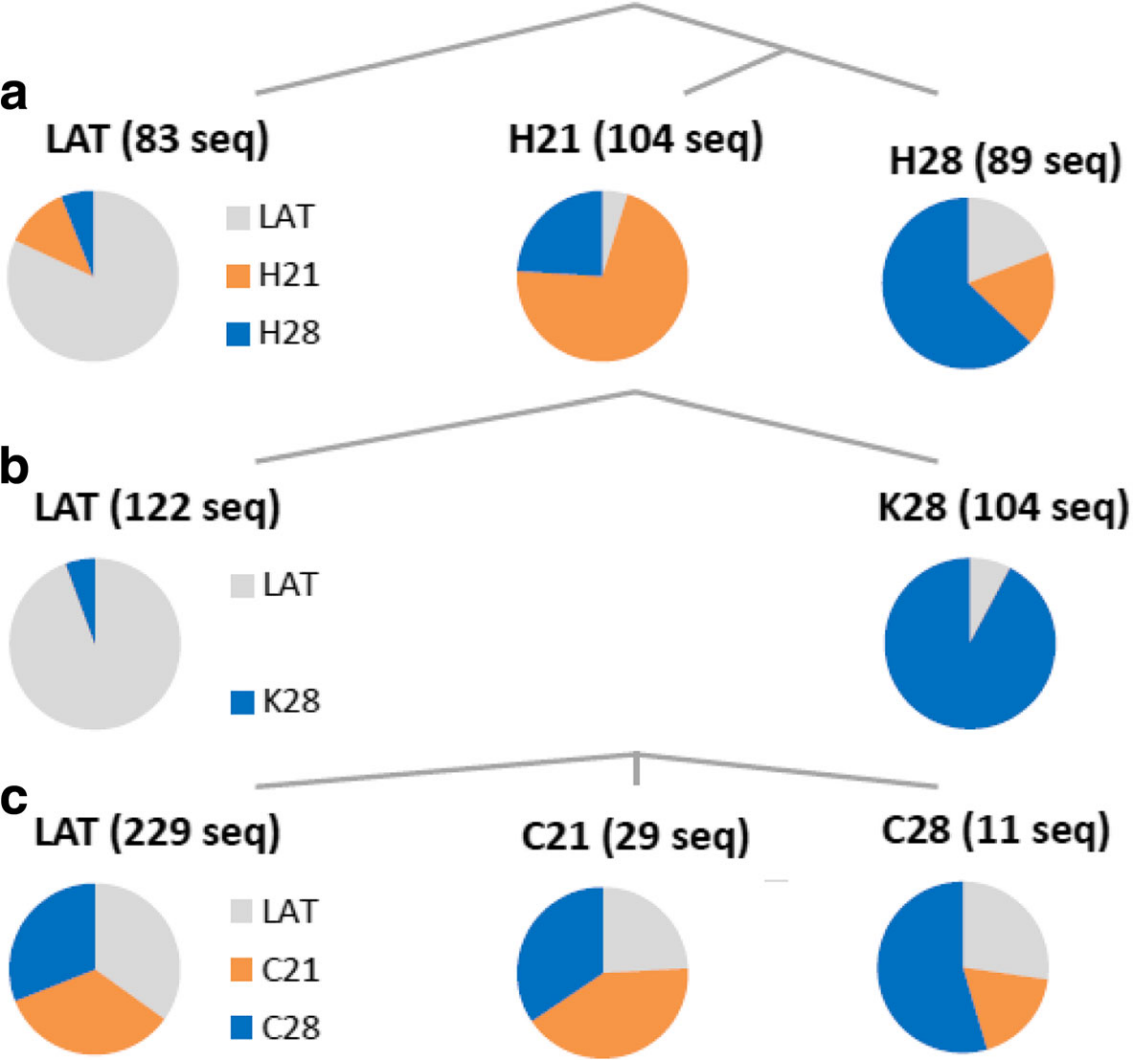
Phylogenetic analysis of uniquely represented sequences from the NGS data set. The clades of each reconstructed tree are identified to their majority group (Lat inoculum, HCV-infected samples at D21 and/or D28 p.i.). **a** Divergent evolution of viral strains in Lat and HepaRG (H) hosts (H). **b** Divergent evolution of viral strains in Lat and Ktmos1 (K) hosts. **c** Lack of evidence for divergence in C6/36 (**c**) hosts. The pie charts show the proportion of each group in each reconstructed phylogenetic clade

Next, we performed a series of next-generation sequencing (NGS) experiments, in order to understand the evolution dynamics of viral populations within cells over the course of infection, and to support replication within mosquito cells. Extraction of the HCV RNA from the inoculum (Lat strain), and the total RNA from HCV-infected cells at days 21 and 28 p.i. for HepaRG and C6/36 (H21, H28 and C21 and C28, respectively) and at day 28 p.i. for Ktmos1 cells (K28) were performed. After processing the RT-PCR on each sample, the amplicons were sequenced on a 454 GS FLX Instrument (Roche). Figure 5 shows the distribution of nucleotide diversity (Fig. A) and Fig. 6 shows the analysis of diversity by the Tajima’s D statistics. For the HCV strains in HepaRG (Fig. 5a) and Ktmos1 (Fig. 5b) cells, the nucleotide diversity increased and the Tajima’s D increased from negative values to positive values. By contrast, nucleotide diversity of the strains in C6/36 (Fig. 5c) cells remained at constant levels and Tajima’s D did not exhibit the same trend as for HepaRG and Ktmos1.

Then, the phylogenetic analysis of sequences from the NGS data set was performed, and illustrated in the Fig. 6. The clades of each reconstructed tree are identified to their majority group (Lat, H21/H28, K28 and C21/C28). A divergent evolution of viral strains was evidenced between the HCV inoculum (Lat, Fig. 6a and b) and H21/H28 (HepaRG, Fig. 6a) and K28 (Ktmos1, Fig. 6b); conversely there is a lack of evidence for divergence between Lat (Fig. 6c) and C21/C28 (C6/36, Fig. 6c). In HepaRG and *Ae aegypti* cells, we detected a selection for a quasispecies, with an increase of global viral diversity suggesting a structuration of the population with elimination of deleterious mutations (Figs. 5a, b and 6a, b). The evolutionary pattern in *Ae albopictus* is different, showing stability of viral diversity and polymorphism.

## Discussion

Very few data have been published about the replication of HCV in mosquitoes or in any other arthropod, although various species have been experimented [18–20, 35]. For instance, Hassan et al. detected HCV viral genome in the heads and midguts of *Cx. pipiens* from 3 h to 8 days after feeding with HCV infected blood, however no sequencing was done and it remained impossible to demonstrate if this was residual or replicated virus. These last authors concluded that mechanical transmission was possible due to the presence of HCV RNA in the mosquito heads after 6 days, but no proofs of HCV replication was given [35]. More than 12 years ago, we published the first results demonstrating the binding of HCV onto *Aedes* cells. In this last study, we investigated the binding of HCV-positive sera (genotype 1b) and we concluded mosquito cells (from *Aedes pseudoscutellaris*) could bind HCV and allow HCV replication for 28 days [22]. At this time the binding of HCV onto mosquito cells was assessed by HCV RNA detection in cells 2 h after inoculation and in the last wash of these cells [22]. Because of the HCV infectiousness of serum samples is highly dependent on host parameters [36, 37], we used in the present study E1E2-expressing HCVpp and clearly showed their ability to associate with the mosquito cell lines *Aedes aegypti* (Aag-2) and *Aedes albopictus* (C6/36). These results are in agreement with our binding results published in 2001, but this time we used both the *bac*HCVpp and the immunofluoresence in a very specific manner. So far, no study determined the capacity of the mosquito cells to bind HCV E1E2 envelope proteins. The E1E2-expressing HCV pseudo particles were shown to be excellent HCV models to study viral binding at the cell surface [12]. HepaRG and liver primary cells have been described as positive models to bind, incorporate and replicate HCV [25, 26, 36, 38]. The characteristics of several mosquito cell surface receptors have been described, but they remain poorly understood. The association of HCV envelope onto mosquito cells raises some questions about putative HCV receptors on mosquito cells. Glycosaminoglycans (GAGs) have been described to bind the HCV particles in a cooperative fashion to receptors, including the Low Density Lipoprotein receptor (LDL-R) and the tetraspanin-family receptor CD81 [15] [39, 40]. Moreover, a number of studies have implicated cell surface GAGs in the initial binding step of DEN and YF virus to cultured mammalian cells [41–43]. Cell-surface GAGs such as heparan sulfates are present on many cell types and are commonly bound by pathogens as a first step of cell penetration [44]. Mosquito cells appear to have the genetic capability to synthesize GAGs [45] and were shown to possess homologues of low density lipoprotein receptor (LDL-R) and the tetraspanin receptor [46, 47]. Presumably, these are the steps required to initiate infection of mosquito cells. The specificities of both the E proteins and their cellular receptors have not been established in detail to date, and it is not clear whether or not the same cellular receptors exist in vertebrates (other than primates) and in mosquito cells, or if the receptors are conserved in all mosquito tissues [48]. It’s worth noticing that, by using flow cytometry and an anti-CD81 human monoclonal antibody, we did not succeed to show CD81 expression at the surface of *Aedes* cells [22].

Cell lines of the mosquito species *Aedes aegypti* and *Aedes albopictus* are known to replicate diverse viruses belonging to the Flavivirus family [49]. We chose these two cell lines, because these two species of *Aedes* mosquitoes are rapidly expanding their territory around the globe and penetrate the developed countries in Europe, Asia and North America, partly due to ecological changes such as global warming [50, 51]. We focused on the infection protocol according to that published before with HepaRG cells (23, and user guide for HepaRG® culture kit 902 for HCV infection, Biopredic International), demonstrating intracellular HCV RNA detection at days 21 and 28 p.i with concomitant production of enveloped HCV infectious particles. This entire work has then been adapted to the C6/36 mosquito cell line and to the original Ktmos1 cell line, developed in our laboratory (see Additional file 2: Figure S1). From the present work it was possible to conclude that the cells from the mosquitoes *Aedes albopictus* (C6/36 cells) and *Aedes aegypti* (Ktmos1) could be infected by serum-derived HCV particles, as viral RNA (+) was detectable at days 21 and/or 28 p.i. To ascertain such a replication, we performed NGS completed with statistical methods computationally very efficient to study RNA virus evolution [52, 53]. Interestingly, similar results were obtained with both human HepaRG hepatocytes and *Ae aegypti* mosquito cells. One of the major results we got in this study is the selection for a HCV quasispecies in *Aedes aegypti* cells, with an increase of global viral diversity suggesting a structuration of the population with elimination of deleterious mutations. The evolutionary pattern in *Ae albopictus* is different (stability of viral diversity and polymorphism), but all in all, these results strongly suggest that the HCV is capable to replicate, not only within human hepatocytes but also within cells from *Aedes* cells.

In 2011, the first HCV homolog was reported in dogs but subsequent studies showed the virus to be widely distributed in horses, as well [54]. This indicated a wider Hepacivirus host range than previously assumed, and paved the way for identification of rodent, bat and non-human primate Hepaciviruses [55, 56]. An entirely open question remained how long-term endemic transmission of HCV is maintained, and there have been very few attempts to redress the paucity of the available data which could clarify this important issue. In an investigation published in 2007, Pybus and colleagues utilized a combined approach integrating bioinformatics and geographic location analyses to build a spatial database of endemic HCV infections. They demonstrated that this database can be used to geographically compare endemic HCV strains with the range distributions of potential vector species [57]. For HCV, well established routes of transmission comprise blood products, drug addiction, sexual transmission and the nosocomial context. Nevertheless, the origin of a significant fraction of HCV infections in humans today remains unexplained, and in fact none of the conditions listed above apply. Moreover, a high prevalence of HCV infection has been observed in hot, humid regions, such as Egypt and in Southern Japan. At the present time, no viable explanation has been put forward for this geographical distribution of the disease. We hypothesize that possibly arthropods could represent the elusive sanctuary. For instance, mosquitoes may be the most common vector responsible for epidemic disease caused by the Flaviviruses. In the present study we have demonstrated that cell lines from two different mosquitoes, which are rapidly spreading towards developed countries, efficiently associate with recombinant *bac*HCVpp expressing E1E2 envelope proteins, suggesting that HCV amplification may be possible via *Aedes mosquitoes*. More conclusive, we completed this first approach by infection experiments studying the dynamics of viral strains over the course of HCV infection.

## Conclusions

We provide, for the first time, credible experimental evidence that HCV could indeed exist in *Aedes* mosquitoes. Our results suggest that these mosquito species may be bona fide vectors responsible for the hidden HCV epidemic, a discovery with major implications for public health and control strategies. Finally our findings provide the basis for a new and deeper probe into the patterns, prevention and treatments of HCV, including a strong focus on arthropodes in future studies.

## Additional files

Additional file 1: Table S1. Characteristics of HCVs Lat strain used for binding/infection experiments. (DOCX 32 kb)

Additional file 2: Figure S1. Global process of the Ktmos1 cell generation from eggs hatching to the final supracellular structures. (A) Macroscopic picture showing the eggs of *Aedes aegypti* collected from insectary. (B) Large hollow vesicles developing at the cut ends of the larvae fragments. (C) Microscopic examination of the adherent cells and molecular identification of cells and larvae extracts by PCR targeting rDNA ITS: the upper panel shows cells in monolayer; the lower panel indicates species-diagnosis PCR of cellular samples with hollow vesicles (lane 1), adherent cells (lane 2) and “Dome-like” structures (lane 3). HEK 293 cells are the negative control, ground larvae extracts of *Aedes aegypti* bora bora strain are the positive control. The approximate size of the amplified product is 550 pb. (D) Microscopic examination of the hollow vesicles as supracellular structures (D1 and D2). (TIFF 751 kb)

Additional file 3: Figure S2. Fluorescence observation of adherent Ktmos1 cells. The Ktmos1 *Aedes aegypti* cells were grown on thin glass (0,17 mm), 2 chambers LabTek (Nunc). The cells were fixed after different periods of cultivation with 2% PFA for 20 min at 37 °C. After permeabilization by PBS containing 0,1% Triton X100 for 2 min, the nuclei were stained by Hoechst 33,258 (Sigma). Observation was performed on motorized inverted Olympus IE81 microscope using the DIC (Differential Interference Contrast) and the DAPI filter. The panel (A) shows a late metaphase stage of a dividing cell. The panel (B) shows Ktmos1 cells in monolayer. (TIFF 925 kb)

Additional file 4: Figure S3. Characteristics of the *bac*Flu-VLPs. Panel A Coomassie stained SDS-PAGE of the *bac*Flupps showing the influenzavirus glycoproteins HA0, HA1 and gp139 (panel A). Panels B and C exhibit EM structures of a genuine influenzavirus particle (Courtesy of Rob Ruigrok Université Grenoble Alpes) and Flu-VLPs used in this study, respectively. (TIFF 2540 kb)

Additional file 5: Figure S4. HCV Infection protocols. (A) for human HepaRG hepatocytes (“H”) and (B) for insect cells, Ktmos1 (“K”, *Ae Aegypti*) and C6/36 (“C”, *Ae Albopictus*). The infection was performed using HCVsp, LAT isolate, genotype 3. D, day; − before infection; D0, day of infection; D4, D7, D14, D21, D28, days post-infection and medium change. P17, P18, passages 17 and 18. HepaRG®, HepaRG cells from KIT902 (Biopredic International). Over the time, HepaRG and Ktmos1 cells in monolayer became more and more differentiated. (TIFF 300 kb)

Additional file 6: Figure S5. Mutations in the HCV IRES region in HCV-infected cells. (A) Structure of HCV IRES region. (B) Mutations observed in HCV-infected HepaRG (H), Ktmos1 (K) and C6/36 (C) cells at days 21 (H21, C21) and 28 (H28, K28, C28) p.i. in the IRES region of the HCV genome. Sequencing by the Sanger method of amplification long products. (TIFF 847 kb)

Additional file 7: Figure S6. Absence of HCV RNA detection in HEK 293 cells. Cells were collected at days 0 (D0), 4 (D4), 21 (D21) and 28 (D28) p.i. The inoculum HCVsp (LAT isolate, genotype 3) was used as positive control. Non-infected (mock) cells (−) and HCV-infected (+) HEK 293 cells. (TIFF 1236 kb)

## Abbreviations

*bac*Flupp: baculovirus-derived Influenza Virus pseudo particles
*bac*HCVpp: baculovirus-derived HCV pseudo particles
GAG: Glycosaminoglycans
HCV-LP: HCV- Like particles
HCVpp: HCV pseudo particles
HCVsp: HCV serum particles
LDL-R: Low density lipoprotein- receptor
NGS: New generation sequencing
