# Supplementary material for: New insights into HCV replication in original cells from Aedes mosquitoes
Source: Virol J. 2017 Aug 22;14:161. doi: 10.1186/s12985-017-0828-z (PMC5567567; doi:10.1186/s12985-017-0828-z)
Supplement: Supplementary file 1 — Characteristics of HCVs Lat strain used for binding/infection experiments. (DOCX 32 kb) [file 12985_2017_828_MOESM1_ESM.docx]

**Supporting Information**

**Generation and observation of the original Ktmos1 *Aedes aegypti* cells**

Primary cultures were prepared using protocols derived several decades ago (Singh 1972). In brief, fifty *Aedes aegypti* (Bora Bora) mosquito eggs collected from the insectary were surface sterilized using 2.6 % sodium hypochlorite and washed two times in 1% PBS (Fig S1A). The neonate larvae were finely chopped with sterile scissors in a 2 mL Eppendorf tubes containing 1 mL L-15 Leibovitz growth medium (Gibco) supplemented with 20% fetal bovine serum, 1% penicillin- Streptomycin, 1% L-glutamine. The cultures were incubated as static cultures in a 28°C incubator and observed using an inverted microscope for tissue attachment and proliferation. This new mosquito cell line has undergone > 100 passages *in vitro*, and has been named Ktmos1. We will notice that two weeks after the cultures were set up, large hollow vesicles developed the cut ends of the tissue fragments and continued to increase in size and number (Fig S1B). These vesicles appeared to consist of monolayers of epithelium-like cells. At this stage, few cells were found attached to surface of 48-wells plates (Fig S1C, upper panel). Three months later, floating tissue fragments with huge hollow vesicles blewup and have been seeded into new wells. Large proportion of the cell masses have attached to the platic. Microscope observations showed that cells were organized in dome-like shape after one month that became new hollow vesicles after 2 months in adherent conditions (Fig S1D). Finally we performed a species-diagnostic PCR by using primers (18SFHIN : 5'GTA AGC TTC CTT TGT ACA CAC CGC CCG T3' ; aeg.r1 : 5' TAA CGG ACA CCG TTC TAG GCC CT 3') designed on the internal transcribed spacer (ITS) regions of the ribosomal DNA (Fig S1C, lower panel). After 55 passages, cells were observed as isolated cells or monolayers. At this stage, cells appeared mononucleated with either an elongated shape or a large square flat cytoplasm (Fig S2A). Mitosis events can sometimes be found in and showed clearly at the late metaphase stage three pairs of chromosomes segregating from the mitotic spindle (Fig 2B)

**Table 1: Characteristics of HCVs Lat strain used for binding/infection experiments**

**
