# Supplementary figures and images for: New insights into HCV replication in original cells from Aedes mosquitoes
Source: Virol J. 2017 Aug 22;14:161. doi: 10.1186/s12985-017-0828-z (PMC5567567; doi:10.1186/s12985-017-0828-z)

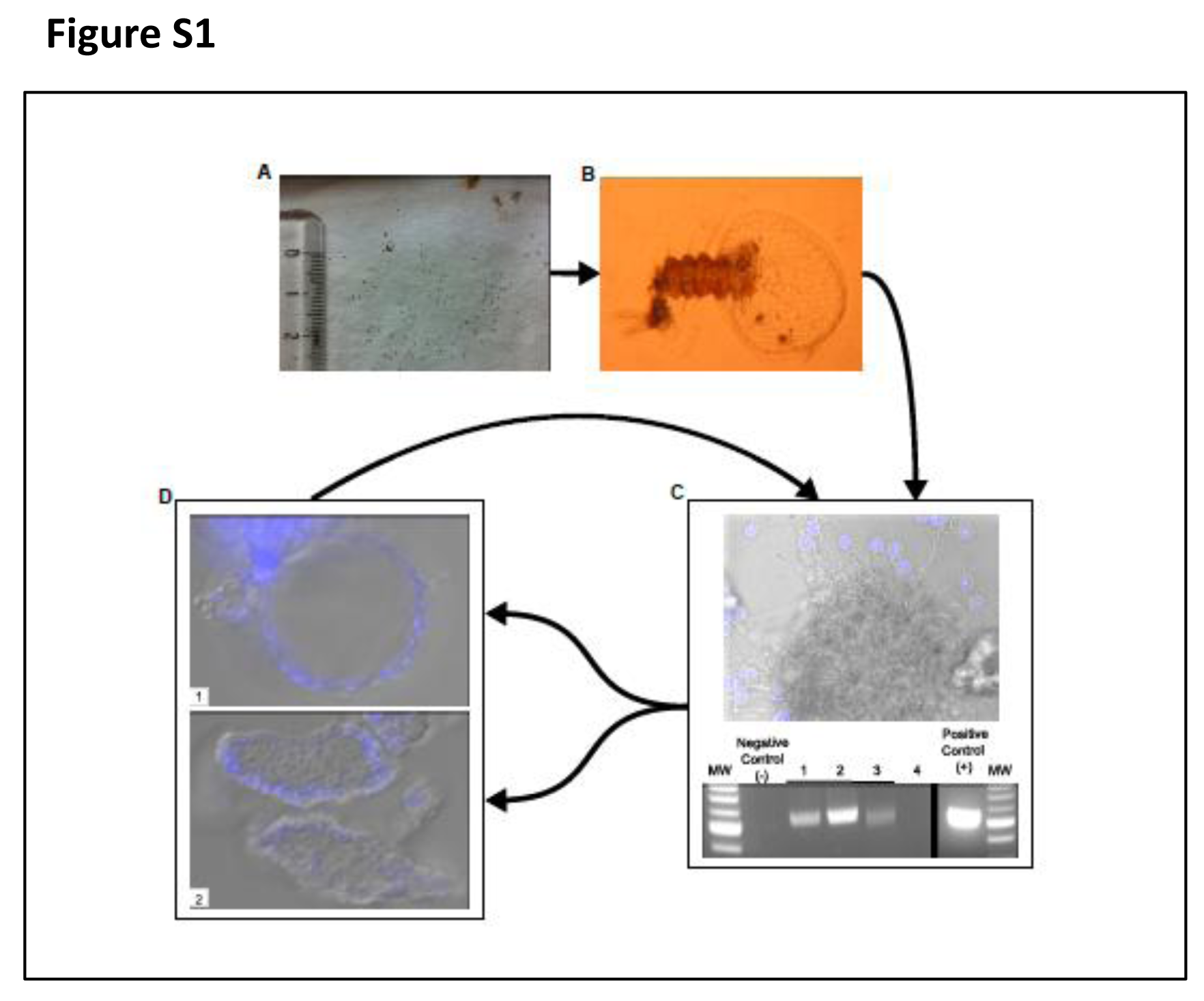

Supplement: Supplementary file 2 — Global process of the Ktmos1 cell generation from eggs hatching to the final supracellular structures. (A) Macroscopic picture showing the eggs of Aedes aegypti collected from insectary. (B) Large hollow vesicles developing at the cut ends of the larvae fragments. (C) Microscopic examination of the adherent cells and molecular identification of cells and larvae extracts by PCR targeting rDNA ITS: the upper panel shows cells in monolayer; the lower panel indicates species-diagnosis PCR of cellular samples with hollow vesicles (lane 1), adherent cells (lane 2) and “Dome-like” structures (lane 3). HEK 293 cells are the negative control, ground larvae extracts of Aedes aegypti bora bora strain are the positive control. The approximate size of the amplified product is 550 pb. (D) Microscopic examination of the hollow vesicles as supracellular structures (D1 and D2). (TIFF 751 kb) [file 12985_2017_828_MOESM2_ESM.tif]

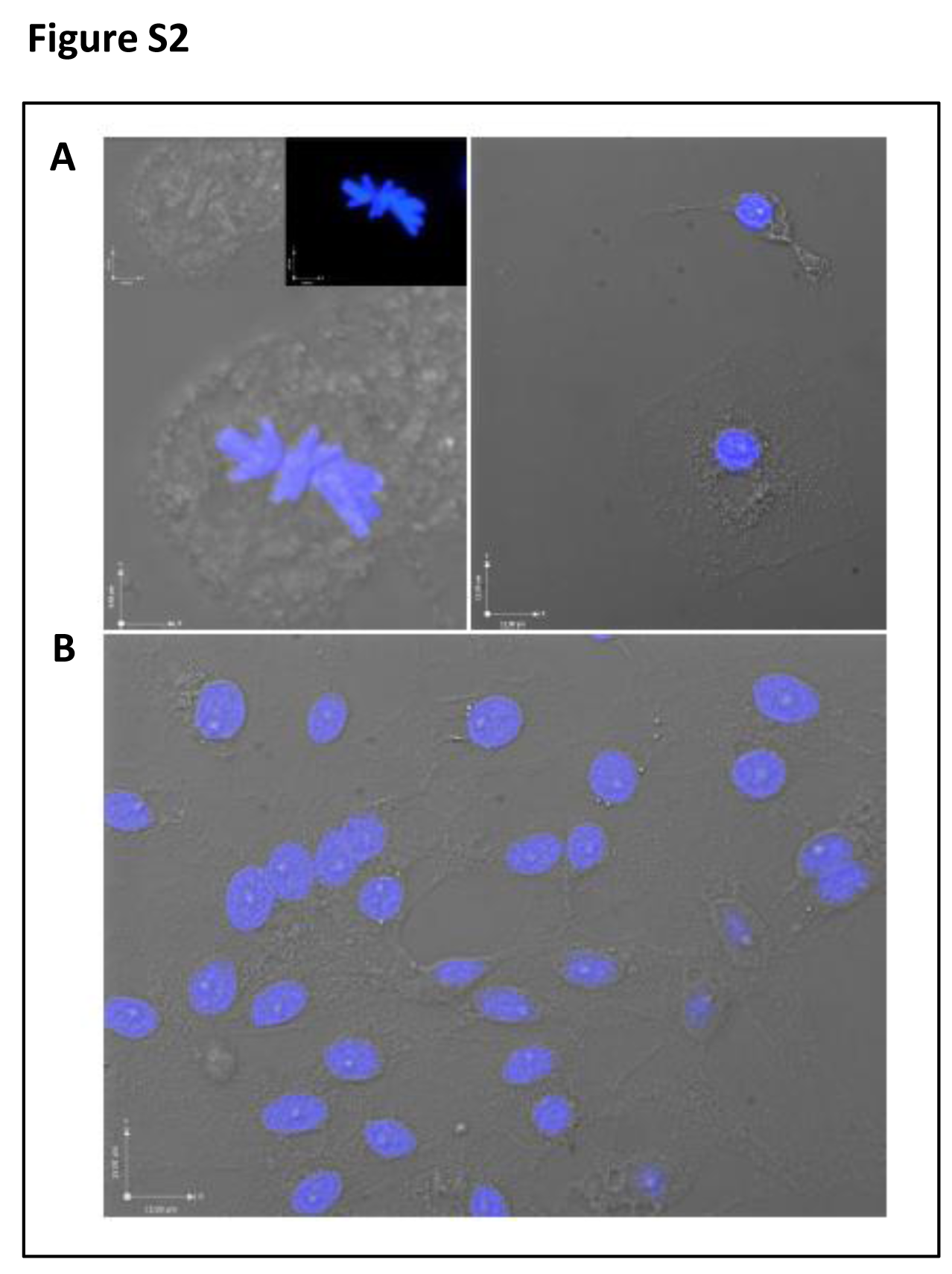

Supplement: Supplementary file 3 — Fluorescence observation of adherent Ktmos1 cells. The Ktmos1 Aedes aegypti cells were grown on thin glass (0,17 mm), 2 chambers LabTek (Nunc). The cells were fixed after different periods of cultivation with 2% PFA for 20 min at 37 °C. After permeabilization by PBS containing 0,1% Triton X100 for 2 min, the nuclei were stained by Hoechst 33,258 (Sigma). Observation was performed on motorized inverted Olympus IE81 microscope using the DIC (Differential Interference Contrast) and the DAPI filter. The panel (A) shows a late metaphase stage of a dividing cell. The panel (B) shows Ktmos1 cells in monolayer. (TIFF 925 kb) [file 12985_2017_828_MOESM3_ESM.tif]

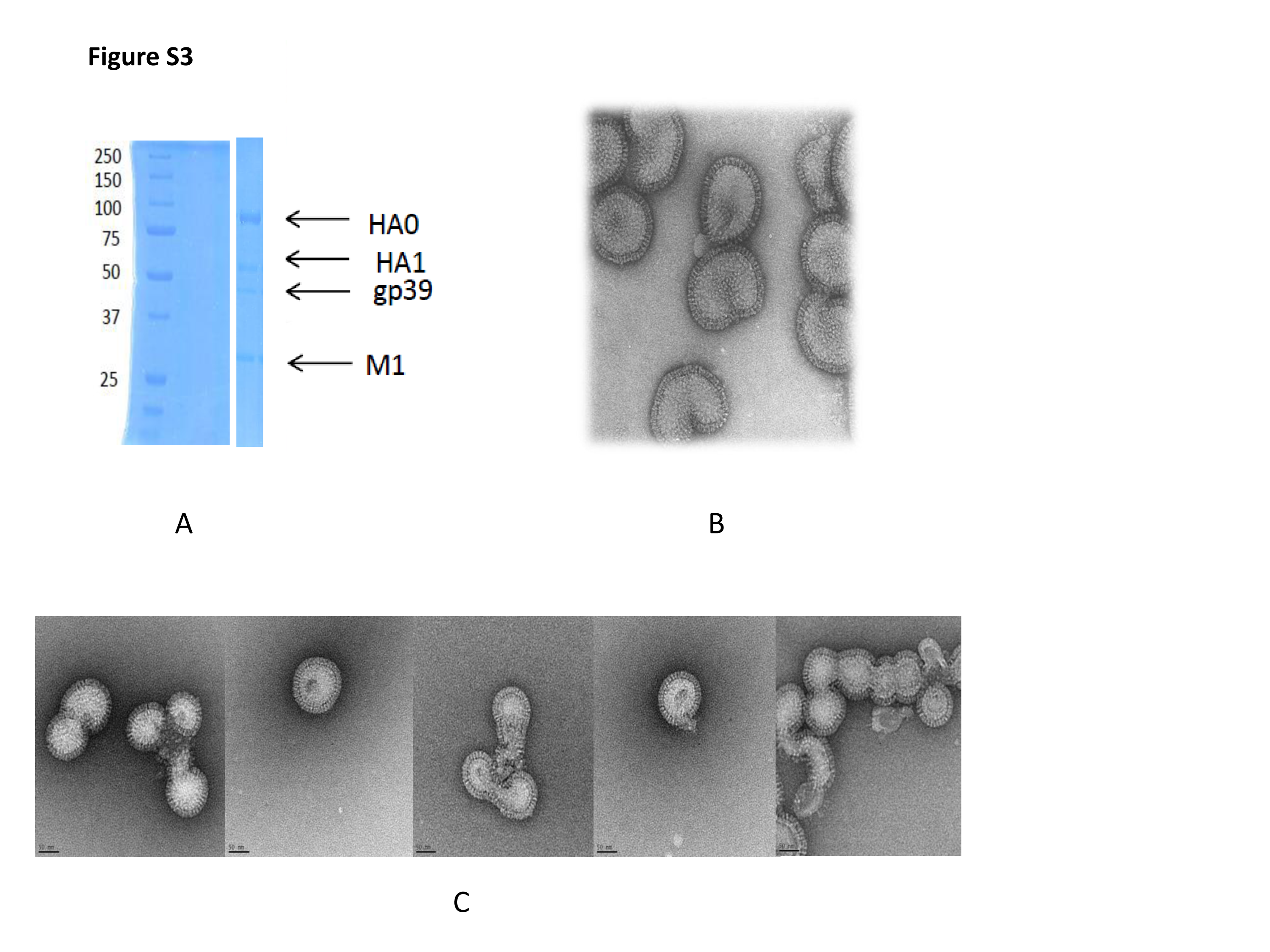

Supplement: Supplementary file 4 — Characteristics of the bacFlu-VLPs. Panel A Coomassie stained SDS-PAGE of the bacFlupps showing the influenzavirus glycoproteins HA0, HA1 and gp139 (panel A). Panels B and C exhibit EM structures of a genuine influenzavirus particle (Courtesy of Rob Ruigrok Université Grenoble Alpes) and Flu-VLPs used in this study, respectively. (TIFF 2540 kb) [file 12985_2017_828_MOESM4_ESM.tif]

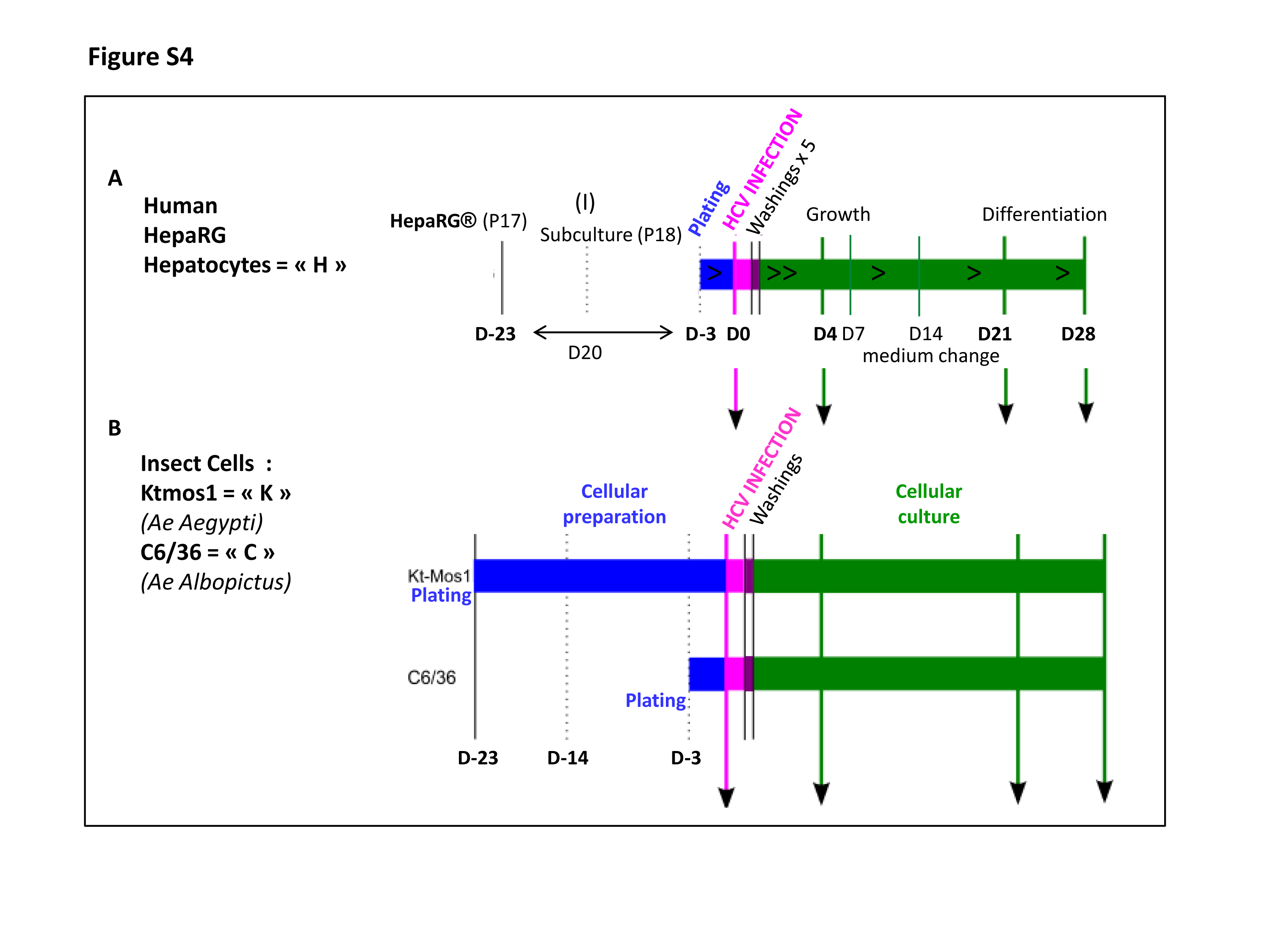

Supplement: Supplementary file 5 — HCV Infection protocols. (A) for human HepaRG hepatocytes (“H”) and (B) for insect cells, Ktmos1 (“K”, Ae Aegypti) and C6/36 (“C”, Ae Albopictus). The infection was performed using HCVsp, LAT isolate, genotype 3. D, day; − before infection; D0, day of infection; D4, D7, D14, D21, D28, days post-infection and medium change. P17, P18, passages 17 and 18. HepaRG®, HepaRG cells from KIT902 (Biopredic International). Over the time, HepaRG and Ktmos1 cells in monolayer became more and more differentiated. (TIFF 300 kb) [file 12985_2017_828_MOESM5_ESM.tif]

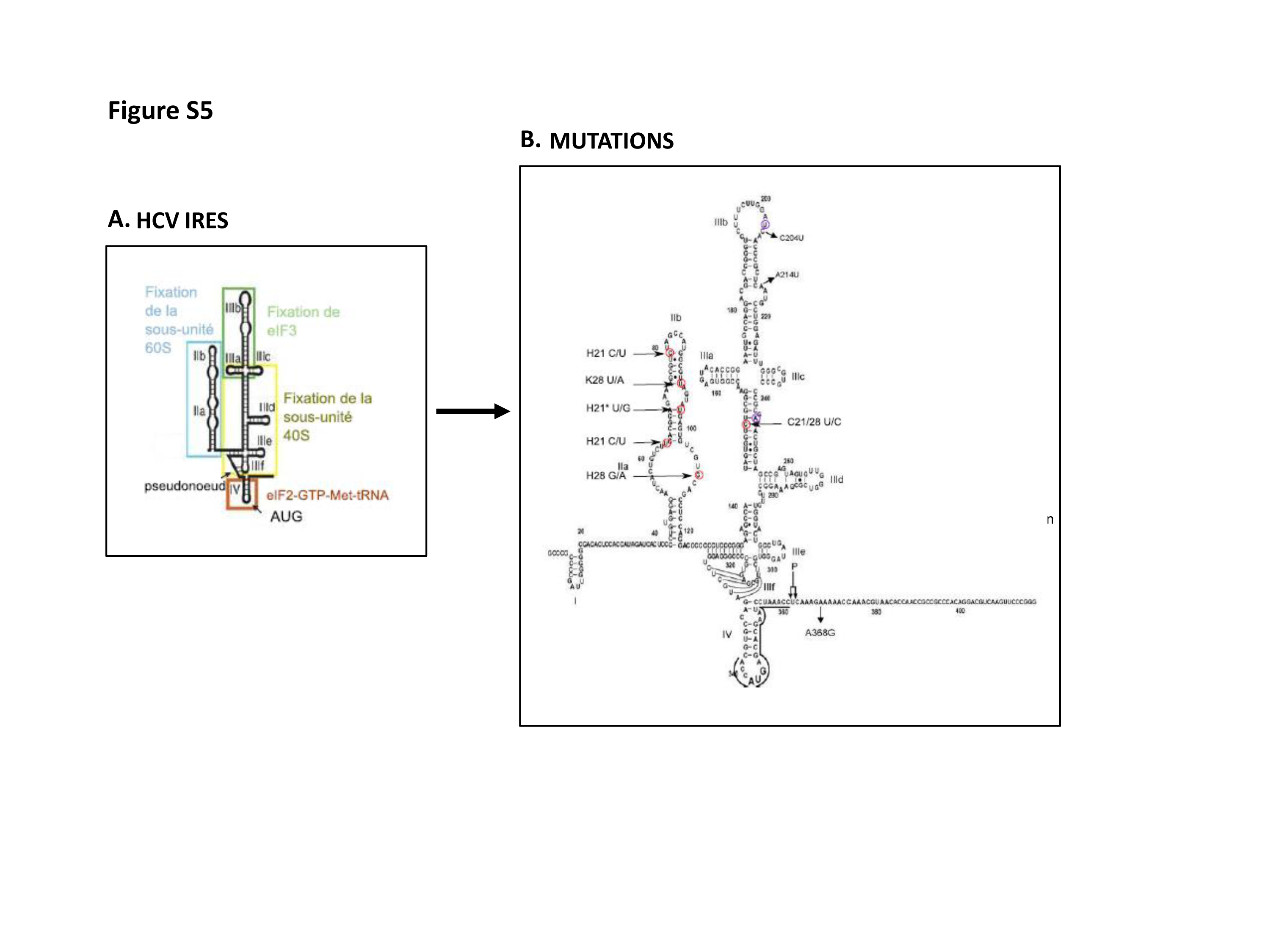

Supplement: Supplementary file 6 — Mutations in the HCV IRES region in HCV-infected cells. (A) Structure of HCV IRES region. (B) Mutations observed in HCV-infected HepaRG (H), Ktmos1 (K) and C6/36 (C) cells at days 21 (H21, C21) and 28 (H28, K28, C28) p.i. in the IRES region of the HCV genome. Sequencing by the Sanger method of amplification long products. (TIFF 847 kb) [file 12985_2017_828_MOESM6_ESM.tif]

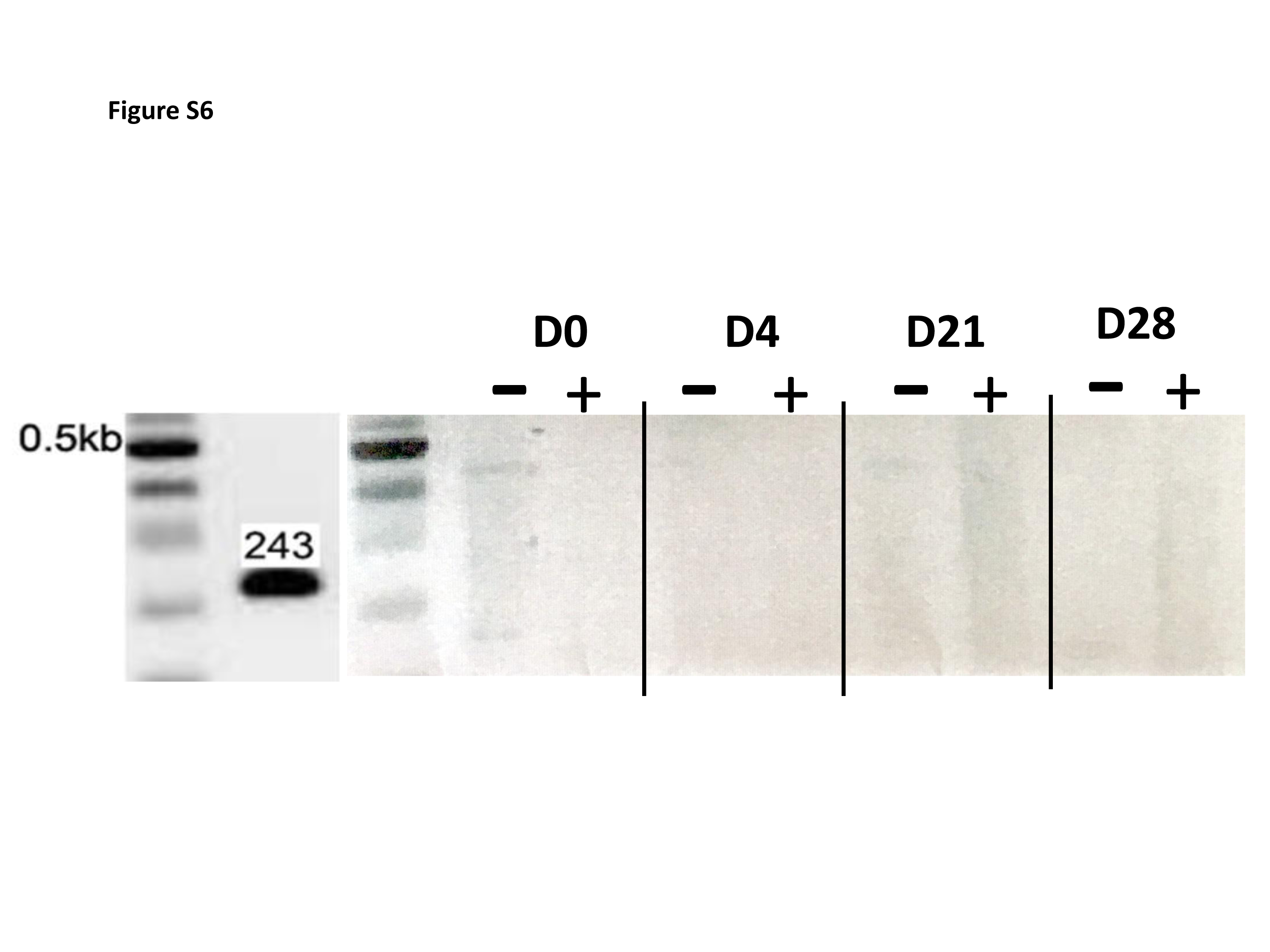

Supplement: Supplementary file 7 — Absence of HCV RNA detection in HEK 293 cells. Cells were collected at days 0 (D0), 4 (D4), 21 (D21) and 28 (D28) p.i. The inoculum HCVsp (LAT isolate, genotype 3) was used as positive control. Non-infected (mock) cells (−) and HCV-infected (+) HEK 293 cells. (TIFF 1236 kb) [file 12985_2017_828_MOESM7_ESM.tif]
